# Ovary Metal Toxicity Remediation by Agro-Food Waste: Evidence for a Regulatory Mechanism of Oxidative Stress by Banana (*Musa cavendish*) Peel Extract

**DOI:** 10.3390/antiox14091129

**Published:** 2025-09-18

**Authors:** Boma F. Eddie-Amadi, Rubina Vangone, Valeria Guerretti, Harrison A. Ozoani, Kenneth O. Okolo, Dokubo Awolayeofori, Tamuno-Boma Odinga-Israel, Kpobari W. Nkpaa, Emidio M. Sivieri, Orish E. Orisakwe, Giulia Guerriero

**Affiliations:** 1 African Centre of Excellence for Oilfield Chemicals Research, University of Port Harcourt, Choba 500102, Nigeria; bamadi17@gmail.com; 2 Comparative Endocrinology Laboratory (EC*lab*), Department of Biology, University of Naples Federico II, 80126 Naples, Italy; vangoneeclab@gmail.com (R.V.); valeria.guerretti@unina.it (V.G.); sivieri.eclab@gmail.com (E.M.S.); 3 African Centre of Excellence for Public Health and Toxicological Research, University of Port Harcourt, Choba 500102, Nigeria; ozoani.harrison@esut.edu.ng (H.A.O.); nkwilly@gmail.com (K.W.N.); 4 Department of Pharmacology & Toxicology, Faculty of Pharmacy, Enugu State, University of Science & Technology, 042, Enugu 400001, Nigeria; kenneth.okolo@esut.edu.ng; 5 Department of Biochemistry, Faculty of Science, Rivers State University, Port Harcourt, Diobu 500264, Nigeria; dokubo.awolayeofori@ust.edu.ng (D.A.); tamuno-boma.odinga@ust.edu.ng (T.-B.O.-I.); 6 Advanced Research Centre, European University of Lefke, Lefke, Northern Cyprus, TR-10, Mersin 99780, Turkey; 7 Interdepartmental Research Center for the Environment (IRCEnv, CIRAm), Via Tarsia 31, 80135 Napoli, Italy

**Keywords:** agro-food waste valorization, antioxidant enzyme activity, banana peel extract, gonadal biomarkers, ovarian histology, oxidative stress markers, phytochemical compounds, pro-inflammatory, apoptotic and transcriptional factors, reproductive hormones, reproductive toxicology

## Abstract

Banana (*Musa cavendish*) peel, usually discarded as waste, is a polyphenol-rich source with antioxidant and chelating properties. This study evaluated its ability to mitigate ovarian toxicity induced by a heavy metal mixture (HMM) consisting of Hg, Mn, Pb, and Al in female rats. Animals received the HMM with or without banana peel extract at 200, 400, and 800 mg/kg dosages for 60 days. Co-treatment dose-dependently reduced ovarian metal accumulation, attenuated oxidative and nitrosative stress (MDA, NO), restored antioxidant enzyme activities (SOD, CAT, GSH, GPx), and modulated pro-inflammatory (IL-6, TNF-α), apoptotic (Caspase-3), and transcriptional factors (NF-κB, Nrf2). The gonadal endocrine profile also improved gonadotropins (FSH, LH), prolactin (PRL), and progesterone (P), which were normalized at the medium dose (400 mg/kg), and demonstrated a clear dose-related effect. Histological examination further revealed that this dose most effectively improved ovarian tissue. GC–MS analysis identified bioactive compounds including resveratrol, proanthocyanidins, and anthocyanidins, supporting both antioxidant and chelating actions. These findings demonstrate that banana peel extract exerts a dual, dose-dependent protective role in the gonad, limiting metal burden while enhancing redox defenses, and highlight its translational potential as a sustainable agro-food waste product in reproductive toxicology.

## 1. Introduction

Metal toxicity is a global problem affecting health, growth, lifespan [1,2,3] and reproductive performance in both humans and animals [4,5,6,7,8]. Among these metals, lead (Pb) and mercury (Hg) are classified as toxicants of major public health concern by the World Health Organization (WHO) [9]. They belong to the class of endocrine-disrupting chemicals (EDCs) and may exert deleterious effects on the reproductive system by altering hormonal activity [6,10,11]. The main routes of human exposure are drinking water and diet [12,13,14]. Due to their slow elimination, metals bioaccumulate in the reproductive organs, liver, kidneys, and blood [11,15,16]. Previous studies demonstrate that heavy metals induce substantial alterations in uterine and ovarian morphology [16,17,18]. Steroidogenesis is particularly vulnerable, and its disruption may lead to ovarian necrosis and functional failure [11,12]. The potential for metal toxicity to cause infertility, especially among women of reproductive age, is therefore of major concern [11,19,20]. Oxidative stress is increasingly recognized as a central mechanism in these reproductive processes. Several studies have shown that it precedes and contributes to hormonal dysfunction by damaging granulosa cells, impairing folliculogenesis, and disrupting steroidogenesis [21,22]. By depleting key antioxidant enzymes such as superoxide dismutase (SOD), catalase (CAT), and glutathione peroxidase (GPx), metals create a redox imbalance that triggers endocrine disruption [23,24]. For this reason, oxidative stress biomarkers are considered early and sensitive indicators of ovarian injury, often preceding measurable hormonal alterations. Importantly, oxidative stress does not act in isolation. Downstream responses involving pro-inflammatory cytokines (interleukin-6, IL-6; tumor necrosis factor alpha, TNF-α), apoptotic mediators (Caspase-3, Cas-3; Bcl-2-associated X protein, Bax), and transcriptional regulators such as nuclear factor kappa B, NF-κB, and factor erythriod 2-related factor 2, Nrf2, are critical in determining the severity of gonadal injury or the activation of adaptive responses [25,26,27]. Investigating these pathways provides a more comprehensive mechanistic understanding of ovarian damage and potential interventions. In recent years, natural compounds have been investigated as protective agents against reproductive toxicity. Plant-derived polyphenols, in particular, have been shown to restore hormonal balance, reduce oxidative stress, and improve ovarian architecture [25,26,27,28,29]. Several of these bioactives are found in agro-food by-products, aligning health protection with sustainability goals [30,31]. This approach reflects the circular bioeconomy concept, in which waste is repurposed as a valuable resource for healthcare applications. Banana (*Musa cavendish*) peel represents a compelling example. It is one of the most abundant fruit wastes worldwide and a rich source of polyphenols such as resveratrol, proanthocyanidins, and anthocyanidins, with well-documented antioxidant and metal-chelating activities [32,33]. Banana peel (BP) extract has been shown to modulate redox-sensitive signaling pathways including Nrf2 and HO-1 and to exert anti-inflammatory and anti-apoptotic effects in vivo [34]. Although banana peel has been traditionally used for cardiovascular conditions [33], its potential in reproductive toxicology has not been explored in studies associated with metals mixture exposure. Most toxicological studies have focused on single metals, whereas real-life exposure typically involves mixtures. Combined exposure can produce synergistic toxicity, particularly in reproductive organs, which are highly susceptible to redox imbalance [5]. To date, no study has evaluated whether BP can mitigate ovarian injury caused by mixed exposure to Pb, Hg, Mn, and Al. Furthermore, it remains unknown whether such protective effects extend simultaneously to biochemical endpoints (oxidative stress, pro-inflammatory, apoptotic, and transcriptional factors) and functional outcomes (hormonal balance and tissue integrity).

This study was designed to fill these gaps. We hypothesized that BP, owing to its dual antioxidant and chelating activities, would reduce ovarian metal burden, regulate oxidative stress, and preserve gonadal endocrine function. In addition, we aimed to demonstrate that agro-food waste can be harnessed as a sustainable bio-resource for reproductive protection. By integrating oxidative stress markers, inflammatory, apoptotic, and transcriptional factors with endocrine and histological evaluation, our work provides new mechanistic evidence for the regulation of ovarian oxidative stress and highlights the translational novelty of banana peel extract in reproductive toxicology.

## 2. Materials and Methods

### 2.1. Preparation of Banana Peels Extracts

Ripped banana fruits were purchased from the International Institute of Tropical Agriculture (IITA) Ibadan and identified by IITA, Ibadan, Oyo State, Nigeria. The peels were thoroughly washed and dried at room temperature, pulverized and then passed through a 60-mesh sieve (approximately 250 μm particle size). Then, 100 g of banana peel powder was weighed and added to 500 mL of deionized water, and then mixed and stored in a refrigerator [34]. Quantification analysis was carried out and 200 mg of banana peel extract was given per kg of animal body weight to a low-dose treatment group, while 400 mg banana peel extract was given per kg of body weight to a medium-dose treatment group and 800 mg banana peel extract was given per kg of body weight to a high-dose treatment group.

### 2.2. Analysis of the Phytochemical Component of the BP Sample by Gas Chromatography

BP preparation for phytocomponent analysis was performed by Gas Chromatography–Mass Spectrometry (GC-MS). GC-MS analysis of the BP methanol extract was performed using the Thermo/Finnigan Surveyor System. Mass spectrometric data were evaluated using data analysis software (Xcalibur Qual Browser 3.1; Thermo Electron, San Jose, CA, USA). Sample preparation and chromatographic separation were carried out following the method reported by Eddie-Amadi et al. [34].

### 2.3. Animals and Treatments

Fifty female albino rats aged 6–8 weeks were purchased from the Pharmacology Animal House, University of Port Harcourt, Rivers State. The animals were housed in standard polypropylene cages under room temperature at 25 ± 2 °C with 12 h light/dark cycles throughout the duration of the experiment. The animals had free access to water and a standard diet. Prior to the commencement of this study, the animals were acclimatized for 1 week. All animal maintenance and experiments were conducted in accordance with the guidelines specified in the protocol sanctioned by the UNIPORT Research Ethics Committee with approval reference number (UPH/CEREMAD/REC/18).

The experimental animals were randomly divided into 5 groups of 10 female rats each (Table 1). The animals received treatment for 60 days as detailed below.

The metal mixtures (Pb 20 mg/kg; Hg 0.40 mg/kg; Mn 0.56 mg/kg; Al 35 mg/kg) were chosen in agreement with models previously published by our group and classified as ‘environmentally relevant—low dose’, since they were derived from environmental monitoring data in the Niger Delta. The doses of banana peel (BP) extract were based on preliminary experiments carried out by Akamine et al. [35] and reported in the work of Eddie-Amadi et al. [34]; the administered doses of banana peel extract were standardized to the dry weight of the pulverized peels used for extraction. Further, the aqueous extract was selected for in vivo administration by oral gavage in order to mimic the traditional/physiological mode of intake and to ensure safety for the experimental animals. 

### 2.4. Sample Collection

After 60 days of treatment, all of the animals in each group were selected for analysis. The selected rats were weighed and anesthetized, and one ovary of each rat was harvested, rinsed in cold saline water, weighed and used for different analyses.

### 2.5. Metal Analysis

The ovaries were immediately isolated, weighed, and subjected to acid digestion according to the work of Hill et al. [36]. Concentrations of lead (Pb), aluminum (Al), mercury (Hg), and manganese (Mn) in ovarian tissue were determined using a Solar Thermo Elemental Flame atomic absorption spectrometer (model SG 71906, Thermo Fisher Scientific, Waltham, MA, USA). The results are expressed in µg/g of tissue. Appropriate blank samples and certified reference standards were included to ensure quality control.

### 2.6. Ovarian Oxidative and Antioxidative Stress Markers

Biomarkers of ovarian oxidative stress were assessed using established spectrophotometric methods reported in the work of Anyachor et al. [37] for the assessment of malondialdehyde (MDA), superoxide dismutase (SOD), catalase (CAT), reduced glutathione (GSH), and glutathione peroxidase (GPx) levels [38] and for nitric oxide (NO) [28]. Malondialdehyde (MDA) reacted with the chromogen in the presence of an acidic medium (2-thiobarbituric acid, TBA), forming a pink complex at 532 nm.

Nitric oxide (NO) levels were estimated by measuring nitrate initially reduced to nitrite using nitrate reductase. Nitrite reacted with sulfanilic acid and N-(1-naphthyl) ethylenediamine under acidic conditions, forming a pink azo dye measurable spectrophotometrically at 540–550 nm.

Superoxide dismutase (SOD) activity was determined by inhibiting O_2_^−^-mediated NADH oxidation at a wavelength of 340 nm for 15 min.

Catalase (CAT) activity was estimated by cleavage of hydrogen peroxide present in the sample at a wavelength of 240 nm.

Reduced glutathione (GSH) levels were estimated by reacting with 5,5′-dithiobis(2-nitrobenzoic acid) (DTNB). GSH reduces DTNB, releasing 5-thio-2-nitrobenzoic acid (TNB), a yellow chromophore measurable spectrophotometrically at 412 nm.

Glutathione peroxidase (GPx) levels were estimated by following the reaction of glutathione reductase and the oxidation of NADPH, with tert-butyl hydroperoxide as the substrate at a wavelength of 340 nm.

### 2.7. Determination of Hormonal Profile Markers

Ovarian concentrations of follicle-stimulating hormone (FSH), luteinizing hormone (LH), prolactin (PRL), and progesterone (P) were quantified using enzyme-linked immunosorbent assay (ELISA) kits (Monobind Inc., Lake Forest, CA, USA; FSH, Cat. No. 425-300 B; LH, Cat. No. 8325-300 E (panel including LH); PRL, Cat. No. 725-300; P, Cat. No. 4825-300 A). The assays were performed according to the manufacturer’s instructions.

### 2.8. Determination of Pro-Inflammatory Factors, Apoptotic and Transcriptional Factors

The activity of interleukin-6 (Il-6; Cat. no.: E-EL-R0015, Elabscience Biotechnology Company, Beijing, China), tumor necrosis factor alpha (TNF-α; Cat no.: RTA00-1, R&D Systems, Elabscience Biotechnology Company, Beijing, China), caspase-3 (Cas-3; Cat. no.: E-EL-R0160, Elabscience), and nuclear factor kappa B (NF-κB; Cat no.: E-ELR0674, Elabscience Biotechnology Company, Beijing, China), and the levels of nuclear factor erythriod 2-related factor 2 (Nrf2; Cat. no.: E-EL-R1052, Elabscience) were measured in the ovaries of rats from both the control and treatment groups using enzyme-linked immunosorbent assay (ELISA) kits.

### 2.9. Histological Analysis

Each ovary was fixed in 10% formaldehyde and then embedded in paraffin and cut into sections with a thickness of 5 μm. The sections were stained with hematoxylin and eosin and examined using a binocular Nikon-MicroPhot-FXA light microscope (Nikon Inc., Melville, NY, USA).

### 2.10. Statistical Analysis

Data were analyzed by one-way analysis of variance (ANOVA) using XLSTAT software (version 2016, Addinsoft, Paris, France). Prior to ANOVA, the assumptions of data normality and homogeneity of variances were verified using the Shapiro–Wilk test and Levene’s test, respectively. These assumptions were met (*p* > 0.05 for all groups). When the overall ANOVA showed significance, post hoc multiple comparisons were performed using Duncan’s multiple range test. Statistical significance was accepted at *p* < 0.05. The results are expressed as mean ± standard deviation (SD). In the graphical charts, groups not sharing the same superscripted letter notations (a-b-c) differ significantly at *p* < 0.05. In addition, exact *p*-values for the main pairwise comparisons (HMM vs. each treatment group) are reported in the corresponding figure legends to ensure transparency and reproducibility.

## 3. Results

### 3.1. Heavy Metal Bioaccumulation in the Ovary of Albino Rat

**Figure 1:**
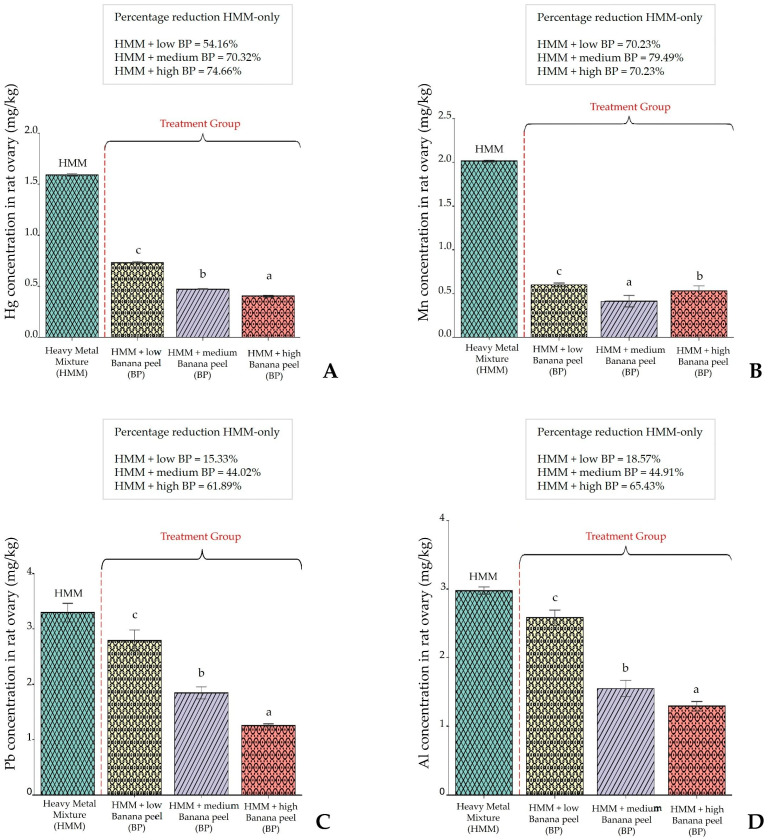
(**A**–**D**) Effect of banana peel (BP) extract on ovarian accumulation of heavy metals in rats co-exposed to a heavy metal mixture (HMM). (**A**) Hg concentration, (**B**) Mn concentration, (**C**) Pb concentration, (**D**) Al concentration. Data are presented as mean ± SD (*n* = 10). Statistical analysis was performed using one-way ANOVA followed by Duncan’s multiple range test. Bars with different letter notations (**a**–**c**) differ significantly at *p* < 0.05. Exact *p*-values for the main comparisons are as follows: **Panel** (**A**) (**Hg**): HMM **vs.** HMM + low BP, *p* = 0.031; HMM vs. HMM + medium BP, *p* = 0.008; HMM vs. HMM + high BP, *p* = 0.002. **Panel** (**B**) (**Mn**): HMM vs. HMM + low BP, *p* = 0.045; HMM vs. HMM + medium BP, *p* = 0.006; HMM vs. HMM + high BP, *p* = 0.012. **Panel** (**C**) (**Pb**): HMM vs. HMM + low BP, *p* = 0.027; HMM vs. HMM + medium BP, *p* = 0.004; HMM vs. HMM + high BP, *p* = 0.001. **Panel** (**D**) (**Al**): HMM vs. HMM + low BP, *p* = 0.039; HMM vs. HMM + medium BP, *p* = 0.009; HMM vs. HMM + high BP, *p* = 0.003.

Figure 1A shows the Hg concentration in albino rat ovaries. The values show a high Hg concentration (1.6 mg/kg) in the HMM-only treated group and a marked reduction in the BP-treated groups. A maximum reduction was observed in the high-BP-dose group with a resultant Hg concentration of 0.4 mg/kg. The reduction compared to the control was dose-dependent. Figure 1B shows the accumulation of Mn, which followed a similar trend. The maximum value for the HMM group was 2.0 mg/kg, and the maximum effect of BP was observed in the medium-BP-dose and high-BP-dose groups. Figure 1B also shows a reduction that is not dose-dependent. Figure 1C shows the accumulation of Pb. BP administration resulted in a marked reduction in the high-dose group. The response was dose-dependent, but the percent reduction was lower than that of Hg and Mn. Figure 1D shows the bioaccumulation of Al and the response to BP treatment. The latter shows a similar trend to that shown for Pb. The response was dose-dependent, with a significant reduction in high-dose BP compared to the control HMM-only group.

### 3.2. Oxidative Stress Markers of Rats Treated with BP Extract Following HMM Exposure

**Figure 2:**
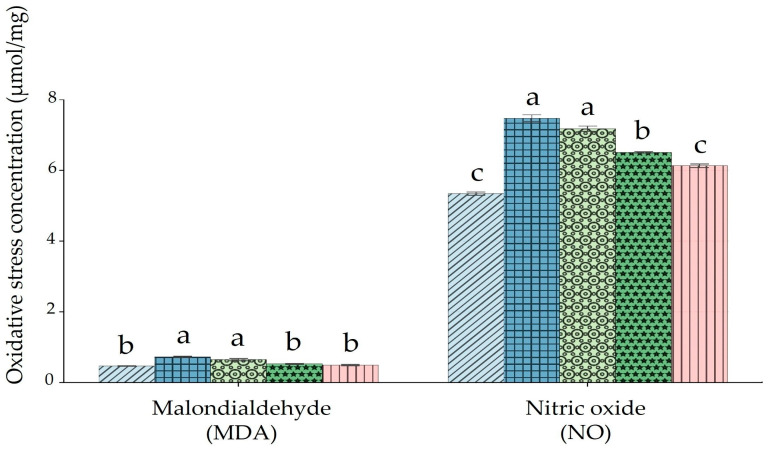
Impact of banana peel extract (BP) on oxidative stress markers in ovary of albino rats exposed for 60 days to a heavy metal mixture (HMM). Effect of BP on malondialdehyde (MDA) and nitric oxide (NO). 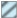 Control; 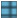 heavy metal mixture (HMM); 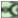 HMM + low banana peel (BP); 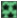 HMM + medium banana peel (BP); 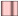 HMM + high banana peel (BP). Values are mean ± SD, *n* = 10. Bars with the same letter notations (a, b, c) are not significantly different from each other (*p* ≥ 0.05). Exact *p*-values for the main pairwise comparisons: **MDA**, HMM vs. HMM + low BP, *p* = 0.042; HMM vs. HMM + medium BP, *p* = 0.011; HMM vs. HMM + high BP, *p* = 0.006; **NO**, HMM vs. HMM + low BP, *p* = 0.027; HMM vs. HMM + medium BP, *p* = 0.004; HMM vs. HMM + high BP, *p* = 0.001.

Ovarian malondialdehyde (MDA) concentrations were significantly higher in the HMM-only and HMM + low-BP groups compared to the control, HMM + medium-BP, and HMM + high-BP groups (Figure 2, MDA). The latter three groups did not differ significantly from each other. Ovarian nitric oxide (NO) levels were significantly higher in the HMM-only and HMM + low-BP groups compared to the control and HMM + high-BP groups (Figure 2, NO). The medium-dose BP group showed intermediate values, significantly lower than the HMM-only group but higher than the control and high-dose BP groups. The high-dose BP group restored NO levels to values not significantly different from those of the control.

### 3.3. Antioxidant Profile of Rats Treated with BP Extract Following HMM Exposure

**Figure 3:**
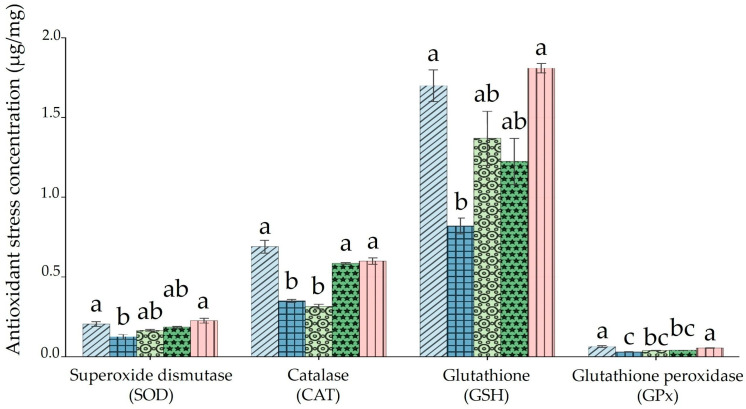
Impact of banana peel extract (BP) on antioxidant profile in ovary of albino rats exposed for 60 days to a heavy metal mixture (HMM). The effect of BP on superoxide dismutase (SOD), catalase (CAT), glutathione (GSH) and glutathione peroxidase (GPx). 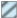 Control; 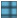 heavy metal mixture (HMM); 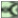 HMM + low banana peel (BP); 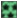 HMM + medium banana peel (BP); 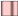 HMM + high banana peel (BP). Values are mean ± SD, *n* = 10. Bars with the same letter notations (a, b, c) are not significantly different from each other (*p* ≥ 0.05). Exact *p*-values for the main pairwise comparisons are as follows: **SOD**, HMM vs. HMM + low BP, *p* = 0.041; HMM vs. HMM + medium BP, *p* = 0.019; HMM **vs.** HMM + high BP, *p* = 0.008; **CAT**, HMM vs. HMM + low BP, *p* = 0.036; HMM vs. HMM + medium BP, *p* = 0.014; HMM vs. HMM + high BP, *p* = 0.006; **GSH**, HMM vs. HMM + low BP, *p* = 0.029; HMM vs. HMM + medium BP, *p* = 0.010; HMM vs. HMM + high BP, *p* = 0.002; **GPx**, HMM vs. HMM + low BP, *p* = 0.048; HMM vs. HMM + medium BP, *p* = 0.021; HMM vs. HMM + high BP, *p* = 0.007.

Superoxide dismutase (SOD) activity was significantly lower in the HMM-only group compared to the control group (Figure 3, SOD). The low- and medium-dose BP groups showed intermediate values, not significantly different from either the control or the HMM group, indicating partial, non-significant improvement. The high-dose BP group restored SOD activity to control levels and these were significantly higher than those of the HMM-only group. Catalase (CAT) activity was significantly reduced in the HMM-only and in the low-dose BP groups compared to the control (Figure 3, CAT). Medium- and high-dose BP groups had values comparable to the control and significantly higher than those of the HMM group. Glutathione (GSH) levels followed a similar pattern: the HMM-only group had significantly lower levels than those of the control (Figure 3, GSH). Low- and medium-dose BP groups were intermediate and not significantly different from either the control or HMM. The high-dose BP group (letter a) was restored to control levels, which were significantly higher than those of the HMM-only group. Glutathione peroxidase (GPx) activity was lowest in the HMM-only group (Figure 3, GPx). The values of the control and high-dose BP groups were significantly higher than those of the HMM.

### 3.4. Phytoconstituents in Banana Peel

Banana peel analysis for phytoconstituents using gas chromatography–mass spectrometry (GC-MS) indicates compounds such as resveratrol, proanthocyanidines, flavonones, delphinidin, pyranpanthiocyanin, aglycone, and malvidin were detected at the following corresponding concentrations: 39.24 μg/mL, 85.48 μg/mL, 20.34 μg/mL, 42.81 μg/mL, 6.81 μg/mL, 25.02 μg/mL and 20.91 μg/mL, respectively.

### 3.5. Hormonal Profile in the Ovary of Rats Treated with BP Extract Following HMM Exposure

**Figure 4:**
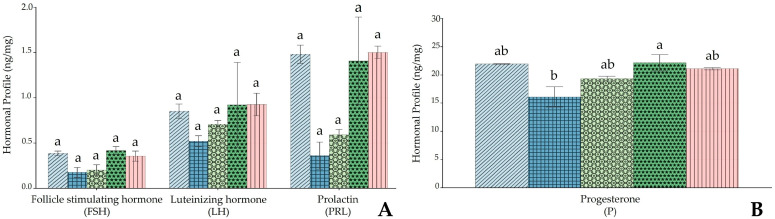
Impact of banana peel extract (BP) on hormonal profile in ovary of albino rats exposed to a heavy metal mixture (HMM) for 60 days. (**A**) Effect of BP on pituitary hormones: follicle-stimulating hormone (FSH), luteinizing hormone (LH) and prolactin (PRL). (**B**) Effect of BP on steroid sex hormone progesterone (P). 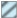 Control; 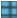 heavy metal mixture (HMM); 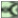 HMM + low banana peel (BP); 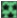 HMM + medium banana peel (BP); 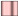 HMM + high banana peel (BP). Values are mean ± SD, *n* = 10. Bars with the same letter notations (a, b) are not significantly different from each other (*p* ≥ 0.05). Exact *p*-values for the main pairwise comparisons are as follows: FSH: HMM vs. HMM + low BP, *p* = 0.112; HMM vs. HMM + medium BP, *p* = 0.089; HMM vs. HMM + high BP, *p* = 0.067; LH: HMM vs. HMM + low BP, *p* = 0.094; HMM vs. HMM + medium BP, *p* = 0.072; HMM vs. HMM + high BP, *p* = 0.058; PRL: HMM vs. HMM + low BP, *p* = 0.081; HMM vs. HMM + medium BP, *p* = 0.065; HMM vs. HMM + high BP, *p* = 0.052; *p*: HMM vs. HMM + low BP, *p* = 0.043; HMM vs. HMM + medium BP, *p* = 0.017; HMM vs. HMM + high BP, *p* = 0.009.

The effects of heavy metal mixture (HMM) exposure and BP treatment on the ovarian hormonal profile are shown in Figure 4A,B.

Follicle-stimulating hormone (FSH), luteinizing hormone (LH), and prolactin (PRL) levels showed differences among the experimental groups, except for progesterone (P). Although the HMM group showed a trend toward reduced FSH, LH, PRL, and P levels compared to the control group, these changes were not statistically significant. Similarly, BP co-treatment at any dose produced increases toward control values, but these improvements did not reach statistical significance.

### 3.6. Banana (Musa cavendish) Peel Extract on Expression of Pro-Inflammatory Factors and Apoptotic and Transcriptional Factors in Male Albino Rat Testis Exposed to HMM

**Figure 5:**
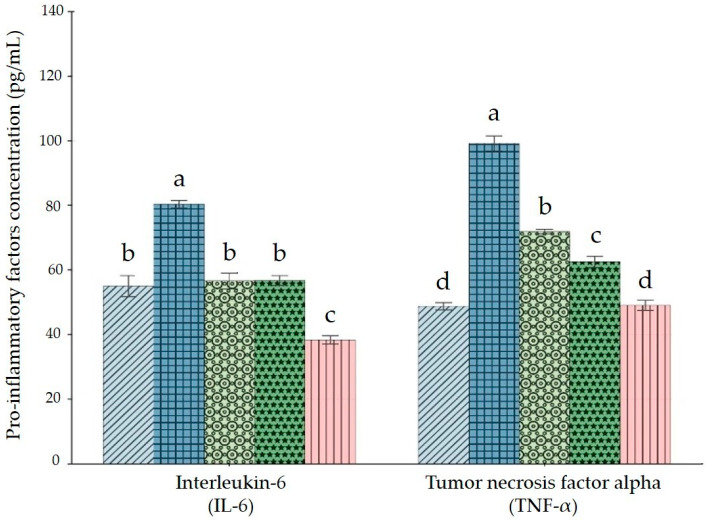
Impact of banana peel extract (BP) on pro-inflammatory factors in ovary of albino rats exposed for 60 days to a heavy metal mixture (HMM). Effect of BP on Interleukin-6 (IL-6) and Tumor necrosis factor alpha (TNF-α). 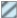 Control; 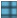 heavy metal mixture (HMM); 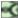 HMM + low banana peel (BP); 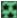 HMM + medium banana peel (BP); 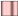 HMM + high banana peel (BP). Values are mean ± SD, *n* = 10. Bars with the same letter notations (a, b, c, d) are not significantly different from each other (*p* < 0.05). Exact *p*-values for the main pairwise comparisons: **IL-6**: HMM vs. Control, *p* = 0.018; HMM vs. HMM + low BP, *p* = 0.027; HMM vs. HMM + medium BP, *p* = 0.021; HMM vs. HMM + high BP, *p* = 0.008. **TNF-α**: HMM vs. Control, *p* = 0.011; HMM vs. HMM + low BP, *p* = 0.023; HMM vs. HMM + medium BP, *p* = 0.015; HMM vs. Hhigh BP, *p* = 0.004.

Exposure to the heavy metal mixture (HMM) resulted in a significant increase in the concentrations of interleukin-6 (IL-6) and tumor necrosis factor-alpha (TNF-α) in the rat ovary compared to the control group (*p* < 0.05). Supplementation with banana peel (BP) attenuated these increases in a dose-dependent manner. In particular, high-dose BP administration led to a significant reduction in IL-6 and TNF-α levels compared to the HMM group (*p* < 0.05), approaching the values observed in the control group (Figure 5).

**Figure 6:**
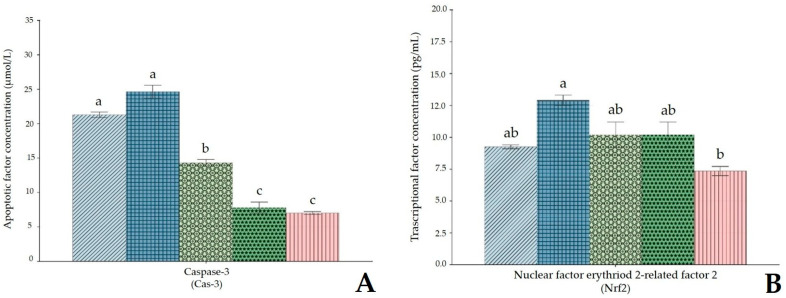
(**A**,**B**) Impact of banana peel extract (BP) on (**A**) apoptotic factor concentration, (B) transcriptional factor concentration in ovary of albino rats exposed for 60 days to a heavy metal mixture (HMM). 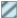 Control; 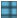 heavy metal mixture (HMM); 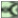 HMM + low banana peel (BP); 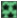 HMM + medium banana peel (BP); 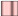 HMM + high banana peel (BP). Values are mean ± SD, *n* = 10. Bars with the same letter notations (a, b, ab, c) are not significantly different from each other (*p* < 0.05). Exact *p*-values for the main pairwise comparisons: (**A**) **Cas-3**: HMM vs. Control, *p* = 0.041; HMM vs. HMM + low BP, *p* = 0.019; HMM vs. HMM + medium BP, *p* = 0.007; HMM vs. HMM + high BP, *p* = 0.004. (**B**) **Nrf2**: HMM vs. Control, *p* = 0.061 (ns); HMM vs. HMM +l ow BP, *p* = 0.047; HMM vs. HMM + medium BP, *p* = 0.039; HMM vs. HMM + high BP, *p* = 0.012.

The effect of HMM and BP on caspase-3 (Cas-3) activity showed a marked increase in caspase-3 levels in the HMM group compared to the control (*p* < 0.05). Co-administration of BP significantly reduced caspase-3 activity, with the most pronounced effect observed in the high-dose BP group (*p* < 0.05) (Figure 6A). Furthermore, exposure to HMM caused a significant decrease in nuclear factor erythroid 2–related factor 2 (Nrf2) levels compared to the control. Supplementation with BP significantly increased Nrf2 levels compared to the HMM group (*p* < 0.05). The protective effect of BP was more pronounced at the high dose, highlighting a potential role in restoring Nrf2 activity (Figure 6B). Exposure to HMM also resulted in a significant increase in nuclear factor kappa B (NF-κB) compared to the control (*p* < 0.05). BP supplementation modulated this response, leading to a significant reduction in NF-κB levels in BP-treated groups compared to the HMM group (*p* < 0.05). The effect was dose-dependent, with values closer to those of the control group observed in rats treated with high-dose BP.

### 3.7. Banana (Musa cavendish) Peel Extract on Histological Profile of the Ovary of Rats Exposed to HMM

**Figure 7:**
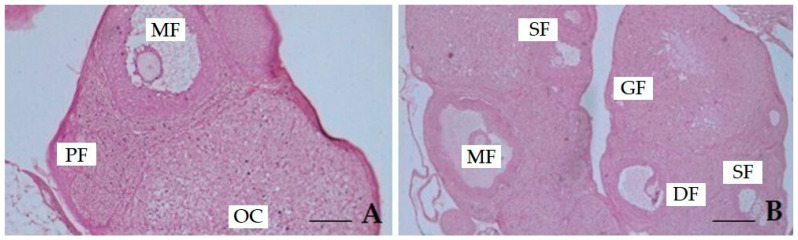
(**A**,**B**)**.** Representative photomicrographs of rat ovaries: (**A**) ovary from the group exposed to heavy metal mixture (HMM), (**B**) ovary from the group co-treated with medium-dose of banana peel extract (BP) (400 mg/kg). ***Abbreviations***: **MF**, mature follicle; **OC**, ovarian cortex/cell deposit; **PF**, primordial follicles; **DF**, degenerating follicles; **GF**, growing follicles; **SF**, secondary follicles. Scale bar = 100 μm.

Ovarian histology from representative rats used in this study is shown in Figure 7A,B.

Ovary micrographs from the HMM-exposed group of rats (Figure 7A) reveal significant morphological alterations in the follicular structures and cortical stroma. Mature follicles (MO) show signs of degeneration, characterized by oocyte disorganization, cytoplasmic vacuolization, and loss of normal antral zone architecture, characteristics indicative of follicular atresia. In the primordial follicle (PF) region, a reduction in cellular density and heterogeneity is observed. These findings indicate early damage to the ovarian reserve. The ovarian cortex (OC) appears disorganized, with areas of reduced cellularity, hypereosinophilic cytoplasm, and pyknotic nuclei, signs that can be attributed to apoptosis or necrosis. Figure 7B shows the ovaries of rats exposed to metals and co-treated with a medium dose of banana peel (BP) extract (400 mg/kg). The micrograph shows follicles at different stages of development, including secondary (SF), growing (GF), and mature (MO) follicles, elements of active folliculogenesis. The granulosa cell layers appear better organized, with reduced signs of cytoplasmic vacuolization and nuclear pyknosis. The ovarian stroma shows a more compact and homogeneous structure. Although degenerating follicles (DF) are still detectable, their frequency is reduced and overall follicular integrity is preserved.

## 4. Discussion

There is growing concern that lifelong exposure to xenobiotics such as heavy metals, in the current era, alters the physiological defense by antioxidants in living organisms, particularly in their reproductive systems [20,39,40]. Heavy metals are endocrine disruptors in animals and humans, and clinical studies have shown that gonads and gametes are susceptible to heavy metal toxicity [19,20,41]. The present study provides the first experimental evidence that banana peel (BP) extract is able to mitigate ovarian injury induced by combined exposure to heavy metals (Pb, Hg, Mn, and Al). In line with our hypothesis, BP exerted protective effects on multiple markers. The novelty of our study lies in two major aspects. First, while most previous investigations have evaluated the protective effects of natural antioxidants against single-metal exposure, we addressed a more realistic scenario, namely, exposure to a mixture of heavy metals, which better reflects human environmental conditions. Second, we identified banana peel, an abundant agro-food by-product, as a sustainable bio-resource capable of restoring ovarian redox homeostasis and transcriptional regulation, thereby providing dual biomedical and environmental value.

### 4.1. Effect of Banana (Musa cavendish) Peel Extract on Bioaccumulation on Ovary Female Albino Rat Exposed to HMM

The accumulation of Hg, Mn, Pb and Al in the ovaries when compared to accumulation in the control rats following exposure to these heavy metals was significantly reduced by BP extract coadministration. Analogous observations of bio metal chelation were reported by Ezejiofor and Orisakwe [42], and Anyanwu et al. [43], albeit with different plant extracts.

### 4.2. Banana (Musa cavendish) Peel Extract Affects Oxidative and Antioxidative Stress Biomarkers of Female Albino Rat Ovary Exposed to HMM

Nitric oxide (NO) normally exerts anti-inflammatory effects under physiological conditions. However, when overproduced through the action of inducible nitric oxide synthase (iNOS), it shifts towards a pro-inflammatory role, driving tissue inflammation. In the present study, NO levels were significantly higher in HMM-exposed rats compared to controls, in agreement with the findings of Nkpaa et al. [44]. Co-treatment with BP extract reduced NO concentrations in a dose-dependent pattern: the medium-dose group showed intermediate levels significantly lower than those of the HMM-only group, while the high-dose group restored NO to values comparable with those of the control. In contrast, changes in malondialdehyde (MDA) were more selective. The HMM-only and low-dose BP groups displayed significantly higher MDA levels than the control, medium, and high-dose BP groups, which did not differ from each other. This suggests that BP at medium and high doses can counteract lipid peroxidation in ovarian tissue, whereas low-dose supplementation was insufficient to normalize MDA. These outcomes point to a dual role of BP extract: attenuation of nitrosative stress (as reflected in NO levels) and mitigation of lipid peroxidation (as reflected in MDA), with the strongest effects seen at higher doses. The observed improvements are consistent with BP’s reported anti-inflammatory activity and its capacity to modulate pro-inflammatory mediators. In addition to influencing these oxidative markers, BP extract demonstrated clear antioxidant activity. HMM exposure led to reductions in superoxide dismutase (SOD), catalase (CAT), glutathione peroxidase (GPx), and glutathione (GSH) activities—key components of the antioxidant defense system [38,45,46,47,48]. This impairment promotes free radical accumulation, membrane lipid damage, and disruption of redox homeostasis. SOD is especially important in detoxifying superoxide radicals and preserving luteal function [49], while GSH directly scavenges lipid peroxides or acts via the GPx–GSH system [50,51,52,53]. The pattern we observed mirrors earlier reports that heavy metals depress antioxidant enzyme activities while elevating oxidative stress markers [5,25,31,45,46,52,53,54,55]. BP co-treatment, particularly at medium and high doses, restored antioxidant enzyme activities and improved GSH content, thereby rebalancing the ovarian redox state. This effect is likely attributable to the extract’s high concentration of polyphenols, including resveratrol and anthocyanidins, which can activate Nrf2 signaling and induce cytoprotective genes such as HMOX-1 [34,56,57]. The phytochemical richness of BP peel—often greater than that of the pulp—reinforces its potential as a dietary strategy against heavy-metal-induced oxidative ovarian damage.

### 4.3. Effect of Banana (Musa cavendish) Peel Extract on Redox-Regulatory Mechanisms Expression of Female Albino Rat Ovary Exposed to HMM

The phytochemical composition of *Musa cavendish* (banana peel, BP) extract includes several bioactive compounds such as resveratrol, proanthocyanidins, flavanones, delphinidin, aglycones, anthocyanidins, and malvidin, most of which belong to the polyphenol family [58]. These molecules are widely recognized for their antioxidant, anti-inflammatory, and anti-apoptotic activities, exerted through the modulation of redox-sensitive signaling pathways and protective proteins [59,60,61,62]. In our study, BP extract restored ovarian antioxidant defenses disrupted by HMM exposure, supporting the evidence that polyphenols can enhance endogenous enzymatic activity (SOD, CAT, GPx, GSH) while limiting lipid peroxidation (MDA) and nitrosative stress (NO) [63,64,65,66,67]. Resveratrol and anthocyanidins, in particular, are known to activate the Nrf2 pathway and induce cytoprotective genes such as HMOX-1 while suppressing NF-κB-mediated transcription of inflammatory cytokines including TNF-α and IL-6 [57,68,69,70,71]. These effects translate into reduced caspase-3 activation and preservation of cellular integrity under oxidative challenge. Notably, anthocyanins such as delphinidin are among the most potent superoxide radical scavengers, reinforcing the antioxidant capacity of BP [72,73]. Emerging evidence also suggests that some polyphenols may influence reproductive hormone regulation by modulating the hypothalamic–pituitary–gonadal axis, contributing to the maintenance of gonadotropins and ovarian steroid balance [45,74]. The phytochemical richness of BP provides a mechanistic explanation for the observed improvements in ovarian redox homeostasis, inflammation, and apoptosis, underscoring its potential as a sustainable bio-resource with reproductive relevance [75,76,77,78,79].

### 4.4. Effect of Banana (Musa cavendish) Peel Extract on Hormonal Profile of the Female Albino Rat Ovary Exposed to HMM

In this work, the analysis of pituitary hormones (FSH, LH, PRL) did not reveal statistically significant differences among the groups, as shown in Figure 4A. This finding contrasts with reports describing marked decreases in LH and FSH after chronic heavy metal exposure [42,43,80] and with the observations of Biswas and Ghosh [81], who reported concomitant reductions in PRL, LH, and FSH. Our results, instead, align more closely with studies by Daku et al. [82] and Riaz et al. [83], in which no significant alterations in gonadotropin levels were recorded. Such variability in outcomes across the literature may reflect differences in the metals involved, the duration of exposure, or the timing of hormone sampling [84]. With regard to ovarian steroids, progesterone levels showed a modest but significant increase in the medium-dose BP group compared with the HMM-only group (Figure 4B). Although the HMM group exhibited a lower progesterone trend than the control, this difference was not statistically significant. The significant improvement observed with medium-dose BP suggests a potential stimulatory action on ovarian steroidogenesis, consistent with the view that certain plant-derived compounds can modulate sex hormone synthesis [43,85,86]. Prolactin concentrations varied among groups but without statistical significance. This outcome differs from some experimental models in which PRL was either elevated or suppressed following heavy metal exposure [81,85,87,88]. Given the heterogeneous nature of PRL responses in previous research, it is plausible that such differences are influenced by factors like sampling time, strain-specific sensitivity, or the particular metals used [54]. These results indicate that, under our experimental conditions, HMM exposure did not induce significant suppression of gonadotropins or marked depletion of progesterone. The selective increase in progesterone with medium-dose BP points toward a possible dose-dependent protective effect, perhaps linked to antioxidant activity and the phytochemical profile of the extract. While the absence of significant changes in most hormones calls for caution in interpretation, it also underscores the need for further work to clarify BP’s potential role in maintaining reproductive endocrine balance under heavy metal stress. A report in progress by our group includes parallel serum and tissue hormonal measurements to provide a more comprehensive understanding of the systemic and local endocrine effects of banana peel extract.

### 4.5. Effect of Banana (Musa cavendish) Peel Extract on Histological Profile of the Female Albino Rat Ovary Exposed to HMM

The histological findings of this study support the biochemical results, confirming that HMM exposure induces severe ovarian injury. Degeneration of mature and primordial follicles, cortical disorganization, and stromal vacuolization reflect the combined action of oxidative stress and inflammation, consistent with previous reports on metal-induced ovarian damage [15,20,39,40,44]. These lesions closely parallel the molecular profile, characterized by elevated IL-6, TNF-α, caspase-3, and NF-κB, together with reduced Nrf2, linking cytokine-driven inflammation and transcriptional dysregulation to follicular atresia and stromal degeneration [25,26,27,34,46]. Co-treatment with BP extract (400 mg/kg) markedly improved ovarian morphology, with better organized granulosa layers and the presence of follicles at different developmental stages indicating resumed folliculogenesis. This recovery mirrors the molecular downregulation of pro-inflammatory and apoptotic markers and the restoration of NF-κB/Nrf2 balance, while the selective rise in progesterone provides an endocrine correlation with histological preservation [42,43,56,57,58,59,80]. Overall, these findings show that BP extract mitigates HMM-induced ovarian toxicity by integrating biochemical, endocrine, and morphological protection [13,34,57,79]. Further work is needed to establish the optimal dose that maximizes tissue protection without adverse effects, but our data clearly indicate that BP represents a promising candidate for mitigating reproductive toxicity associated with heavy metals.

## 5. Conclusions

To the best of our knowledge, this is the first study to show that banana peel (BP) of *Musa cavendish* extract can protect the ovary from heavy metal toxicity by restoring its natural antioxidant defenses. What makes this work distinctive is not only the demonstration of a dual antioxidant–chelating action, but also the idea of giving new life to an agricultural by-product and showing its relevance in a field as sensitive as reproductive health. Our findings indicate that medium and high doses of BP extract significantly reduced oxidative stress and rebalanced ovarian redox homeostasis while at the same time supporting tissue integrity. By connecting the phytochemical richness of BP with measurable improvements in molecular pathways, hormonal activity, and ovarian morphology, this study contributes to a growing body of evidence that natural antioxidants can defend reproductive organs from environmental insults. It also sends a broader message: agricultural waste, often considered valueless, can instead become a sustainable resource with direct implications for women’s health. Future studies will need to clarify the exact molecular mechanisms involved and test whether these protective effects extend to different models of infertility linked to oxidative stress.

## Figures and Tables

**Table 1:** Experimental setup. Amounts added to daily feeds per kilogram of body weight.

Group	Treatment
Group 1 (Control)	Standard diet and deionized water only.
Group 2 (Toxicity Control)	Metal mixture: Pb, (20 mg/kg), Hg (0.40 mg/kg), Mn (0.560 mg/kg) and Al (35 mg/kg)
Group 3	Treated with 200 mg/kg banana peel extract + metal mixture
Group 4	Treated with 400 mg/kg banana peel extract + metal mixture
Group 5	Treated with 800 mg/kg banana peel extract + metal mixture

Number of rats per group = 10.

## Data Availability

Data are contained within this article.

## References

[1] Jomova K., Alomar S.Y., Nepovimova E., Kuca K., Valko M. (2025). Heavy metals: Toxicity and human health effects. Arch. Toxicol..

[2] Panda B.P., Mohanta Y.K., Parida S.P., Pradhan A., Mohanta T.K., Patowary K., Mahari W.A.W., Lam S.S., Ghfar A.A., Guerriero G. (2023). Metal pollution in freshwater fish: A key indicator of contamination and carcinogenic risk to public health. Environ. Pollut..

[3] Adeogun A.E., Ogunleye O.D., Akhigbe T.M., Oyedokun P.A., Adegbola C.A.L., Saka W.A., Afolabi O.A., Akhigbe R.E. (2025). Impact of arsenic on male and female reproductive function: A review of the pathophysiology and potential therapeutic strategies. Naunyn Schmiedebergs Arch. Pharmacol..

[4] Piscopo M., Notariale R., Rabbito D., Ausió J., Olanrewaju O.S., Guerriero G. (2018). *Mytilus galloprovincialis* (Lamarck, 1819) spermatozoa: hsp70 expression and protamine-like protein property studies. ESPR.

[5] Wu K., Chen Y., Huang W. (2025). Combined molecular toxicity mechanism of heavy metals mixtures. Toxicological Assessment of Combined Chemicals in the Environment.

[6] Laoye B., Olagbemide P., Ogunnusi T., Akpor O. (2025). Heavy metal contamination: Sources, health impacts, and sustainable mitigation strategies with insights from nigerian case studies. F1000Research.

[7] Guerriero G., D’Errico G., Di Giaimo R., Rabbito D., Olanrewaju O.S., Ciarcia G. (2018). Reactive oxygen species and glutathione antioxidants in the testis of the soil biosentinel *Podarcis sicula* (Rafinesque 1810). Environ. Sci. Pollut. Res..

[8] Yang Y., Zhang W., Wang S., Zhang H., Zhang Y. (2020). Response of male reproductive function to environmental heavy metal pollution in a free-living passerine bird, *Passer Montanus*. Sci. Total Environ..

[9] Balali-Mood M., Eizadi-Mood N., Moghaddam H.H., Etemad L., Moshiri M., Vahabzadeh M., Sadeghi M. (2025). Recent advances in the clinical management of intoxication by five heavy metals: Mercury, lead, chromium, cadmium and arsenic. Heliyon.

[10] Bhardwaj J.K., Bikal P., Sachdeva S.N. (2024). Cadmium as an ovarian toxicant: A review. J. Appl. Toxicol..

[11] Dutta S., Gorain B., Choudhury H., Roychoudhury S., Sengupta P. (2022). Environmental and occupational exposure of metals and female reproductive health. ESPR.

[12] Witkowska D., Słowik J., Chilicka K. (2021). Heavy metals and human health: Possible exposure pathways and the competition for protein binding sites. Molecules.

[13] Mititelu M., Neacșu S.M., Busnatu Ș.S., Scafa-Udriște A., Andronic O., Lăcraru A.E., Ioniță-Mîndrica C.-B., Lupuliasa D., Negrei C., Olteanu G. (2025). Assessing heavy metal contamination in food: Implications for human health and environmental safety. Toxics.

[14] Fatima G., Raza A.M., Dhole P. (2025). Heavy Metal Exposure and Its Health Implications: A Comprehensive Review. Indian J. Clin. Biochem..

[15] Islam M., Roy D., Singha D. (2025). Metal Ion Toxicity in Human Body: Sources, Effects, Mechanisms and Detoxification Methods. CHAF.

[16] Ali Z., Sher N., Muhammad I., Nayab G.E., Alouffi A., Almutairi M.M., Khan I., Ali A. (2025). The combined effect of cadmium and copper induces bioaccumulation, and toxicity and disrupts the antioxidant enzymatic activities of goldfish (*Carassius auratus*). Toxicol. Rep..

[17] Höfer N., Diel P., Wittsiepe J., Wilhelm M., Degen G.H. (2009). Dose and route dependent hormonal activity of the metalloestrogen cadmium in the rat uterus. Toxicol. Lett..

[18] Wang Y., Wang X., Wang Y., Fan R., Qiu C., Zhong S. (2015). Effect of cadmium on cellular ultrastructure in mouse ovary. Ultrastruct. Pathol..

[19] Srnovršnik T., Virant-Klun I., Pinter B. (2023). Heavy metals and essential elements in association with oxidative stress in women with polycystic ovary syndrome—A systematic review. Antioxid.

[20] He Y., Su X., Niu Z., Zhang B., Mu H., Wang L., Yao Y., Wang X. (2025). Association Between Mixed Metal Exposures and Female Infertility: A Large Cross-sectional Study. IJER.

[21] Agarwal A., Aponte-Mellado A., Premkumar B.J., Shaman A., Gupta S. (2012). The effects of oxidative stress on female reproduction: A review. Reprod. Biol. Endocrinol..

[22] Luderer U. (2014). Ovarian toxicity from reactive oxygen species. Vitam. Horm..

[23] Flora S.J.S., Mittal M., Mehta A. (2008). Heavy metal induced oxidative stress & its possible reversal by chelation therapy. Indian J. Med. Res..

[24] Mishra K.P., Singh V.K., Rani R., Yadav V.S., Chandran V., Srivastava R.K., Tripathi P. (2014). Radiation-induced oxidative stress and its amelioration. J. Environ. Pathol. Toxicol. Oncol..

[25] Ozoani H.A., Orisakwe O.E., Parisi C., Assisi L., Ezejiofor A.N., Okolo K.O., Orish C.N., Vangone R., Sivieri E.M., Guerriero G. (2024). Role of *Anonychium africanum* (plantae, Fabaceae) in metal Oxido-inflammatory response: Protection evidence in gonad of male albino rat. Antioxid.

[26] Ruslee S.S., Zaid S.S.M., Bakrin I.H., Goh Y.M., Mustapha N.M. (2020). Protective effect of Tualang honey against cadmium-induced morphological abnormalities and oxidative stress in the ovary of rats. CAM.

[27] Zaid S.S.M., Othman S., Kassim N.M. (2018). Protective role of *Ficus deltoidea* against BPA-induced impairments of the follicular development, estrous cycle, gonadotropin and sex steroid hormones level of prepubertal rats. J. Ovarian Res..

[28] Haseeb A., Naz S., Satti S., Khan R.U., Asad F., Alrefaei A.F., Almutairi M.H., Momand N.K., Ibiwoye D.I. (2025). Comparative impact of selenium sources and doses on sexual behaviour, productivity and gonadal bioaccumulation in *Coturnix coturnix* japonica. J. Appl. Anim. Res..

[29] Ekaye S.O., Uwagie-Ero E.A., Odigie E.A., Aghayedo C.O. (2018). Protective effect of shea oil on the ovary of albino rats intoxicated with refinery effluents. Anim. Res. Int..

[30] Salehi B., Mishra A.P., Nigam M., Sener B., Kilic M., Sharifi-Rad M., Fokou P.V.T., Martins N., Sharifi-Rad J. (2018). Resveratrol: A Double-Edged Sword in Health Benefits. Biomedicines.

[31] Anyachor C.P., Orisakwe O.E., Orish C.N., Parisi C., Vangone R., Guerretti V., Assisi L., Ajibo D.N., Dooka B.D., Ezealisiji K.M. (2025). Testis metal toxicity remediation by agro-food waste: Evidence of a protective effect of melon seed husk extract *Cucumeropsis mannii* silica nanoparticles on gonadotropin and sex steroid hormones. Environ. Sci. Pollut Res.

[32] Gouw V.P., Jung J., Zhao Y. (2021). Functional properties, bioactive compounds, and in vitro health benefits of banana peel: A review. Food Rev. Int..

[33] Edenta C., James D.B., Owolabi O.A., Okoduwa S.I.R. (2014). Hypolipidemic effects of aqueous extract of three Cultivars of *Musa sapientum* fruit peel on poloxamer-407 induced hyperlipidemic wistar rats. Int. J. Pharm. Sci. Res..

[34] Eddie-Amadi B.F., Ezejiofor A.N., Orish C.N., Rovira J., Allison T.A., Orisakwe E.O. (2022). Banana peel ameliorated hepato-renal damage and exerted anti-inflammatory and anti-apoptotic effects in metal mixture mediated hepatic nephropathy by activation of Nrf2/Hmox-1 and inhibition of Nfkb pathway. Food Chem. Toxicol..

[35] Akamine K., Tomoyuki K., Kazunaga Y. (2009). Banana peel extract suppressed prostate gland enlargement in testosterone-treated mice. Biosci. Biotechnol. Biochem..

[36] Hill A.D., Patterson K.Y., Veillon C., Morris E.R. (1986). Digestion of bio logical materials for mineral analyses using a combination of wet and dry ashing. Anal. Chem..

[37] Anyachor C.P., Orish C.N., Ezejiofor A.N., Cirovic A., Cirovic A., Ezealisiji K.M., Orisakwe O.E. (2023). Nickel and aluminium mixture elicit memory impairment by activation of oxidative stress, COX-2, and diminution of AChE, BDNF and NGF levels in cerebral cortex and hippocampus of male albino rats. Curr. Res. Toxicol..

[38] Guerriero G., DiFinizio A., Ciarcia G. (2002). Stress-Induced Changes of Plasma Antioxidants in Aquacultured Sea Bass, *Dicentrarchus labrax*. Comp. Biochem. Physiol. A Mol. Integr. Physiol..

[39] Thakur S., Chandra A., Kumar V., Bharti S. (2025). Environmental Pollutants: Endocrine Disruptors/Pesticides/Reactive Dyes and Inorganic Toxic Compounds Metals, Radionuclides, and Metalloids and Their Impact on the Ecosystem. Biotechnology for Environmental Sustainability.

[40] Khaksar M.R., Dehchenari R.A., Ghafuri Y. (2025). Characterization and Toxicity Mechanism of Environmental Risk Factors (Heavy Metals) and Reproductive Health: A Review Paper. J. Chem. Health Risks.

[41] Virtuoso S., Raggi C., Maugliani A., Baldi F., Gentili D., Narciso L. (2024). Toxicological effects of naturally occurring endocrine disruptors on various human health targets: A rapid review. Toxics.

[42] Ezejiofor A.N., Orisakwe O.E. (2017). Evaluation of Protective Effect of Aqueous Leave Extract of *Costus afer* onFemale Albino Wistar Rats Exposed to Lead Acetate. EC Pharm Tox..

[43] Anyanwu B.O., Orish C.N., Ezejiofor A.N., Nwaogazie I.F., Orisakwe O.E. (2020). Neuroprotective effect of *Costus afer* on low dose heavy metal mixture (lead, cadmium and mercury) induced neurotoxicity via antioxidant, anti-inflammatory activities. Toxicol. Rep..

[44] Nkpaa K.W., Amadi B.A., Awogbindin I.O., Abolaji A.O., Adedara I.A., Wegwu M.O., Farombi E.O. (2018). Ethanol exacerbates manganese—Induced neurobehavioral defcits, striatal oxidative stress and apoptosis via regulation of p53, caspase-3 and Bax/Bcl-2 ratio-dependent pathway in rat striatum. Biol. Trace Elem. Res..

[45] Fan Y., Jiang X., Xiao Y., Li H., Chen J., Bai W. (2024). Natural antioxidants mitigate heavy metal induced reproductive toxicity: Prospective mechanisms and biomarkers. Crit. Rev. Food Sci. Nutr..

[46] Jomova K., Alomar S.Y., Alwasel S.H., Nepovimova E., Kuca K., Valko M. (2024). Several lines of antioxidant defense against oxidative stress: Antioxidant enzymes, nanomaterials with multiple enzyme-mimicking activities, and low-molecular-weight antioxidants. Arch. Toxicol..

[47] Xiao C.L., Lai H.T., Zhou J.J., Liu W.Y., Zhao M., Zhao K. (2025). Nrf2 signaling pathway: Focus on oxidative stress in spinal cord injury. Mol. Neurobiol..

[48] Guerriero G., Di Finizio A., Ciarcia G. (2003). Oxidative defenses in the sea bass, *Dicentrarchus labrax*. Oxygen Transport to Tissue XXIV.

[49] Dutta S., Sengupta P., Izuka E., Menuba I., Nwagha U. (2024). Oxidative and nitrosative stress and female reproduction: Roles of oxidants and antioxidants. J. Integr. Sci. Technol..

[50] Zhang Z., Shi C., Wang Z. (2023). Therapeutic effects and molecular mechanism of chlorogenic acid on polycystic ovarian syndrome: Role of HIF-1alpha. Nutrients.

[51] Zhang Y., Wang Y., Zhao G., Orsulic S., Matei D. (2023). Metabolic dependencies and targets in ovarian cancer. Pharmacol Ther..

[52] Sun Q., Li Y., Shi L., Hussain R., Mehmood K., Tang Z., Zhang H. (2022). Heavy metals induced mitochondrial dysfunction in animals: Molecular mechanism of toxicity. Toxicology.

[53] Balali-Mood M., Naseri K., Tahergorabi Z., Khazdair M.R., Sadeghi M. (2021). Toxic mechanisms of five heavy metals: Mercury, lead, chromium, cadmium, and arsenic. Front. Pharmacol..

[54] Al-Ani N.K., Al-Kawaz U., Saeed B.T. (2015). Protective Influence of Zinc on Reproductive Parameters in Male Rat Treated with Cadmium. Am. J. Med. Sci..

[55] Jamshidi Z., Roohbakhsh A., Karimi G. (2023). An overview on the protective effects of ellagic acid against heavy metals, drugs, and chemicals. Food Sci. Nutr..

[56] Puraikalan Y. (2018). Characterization of proximate, phytochemical and antioxidant analysis of banana (*Musa sapientum*) peels/skins and objective evaluation of ready to eat/cook product made with banana peels. Curr. Res. Nutr. Food Sci..

[57] Meng T., Xiao D., Muhammed A., Deng J., Chen L., He J. (2021). Anti- Inflammatory Action and Mechanisms of Resveratrol. Molecules.

[58] Mattioli V., Zanolin M.E., Cazzoletti L., Bono R., Cerveri I., Ferrari M., Pirina P., Garcia-Larsen V. (2020). Dietary flavonoids and respiratory diseases: A population-based multi-case–control study in Italian adults. Public Health Nutr..

[59] Gupta G., Saxena S., Baranwal M., Reddy M.S. (2022). In vitro evaluation of bioactive properties of banana sap. Biologia.

[60] Nisar A., Jagtap S., Vyavahare S., Deshpande M., Harsulkar A., Ranjekar P., Prakash O. (2023). Phytochemicals in the treatment of inflammation-associated diseases: The journey from preclinical trials to clinical practice. Front. Pharmacol..

[61] Arijit M., Banerjee S., Bose S., Das P.P., Sandberg E.N., Atanasov A.G., Bishayee A. (2021). Cancer preventive and therapeutic potential of banana and its bioactive constituents: A systematic, comprehensive, and mechanistic review. Front. Oncol..

[62] Ashka F., Dubey P.K., Kumar S., Dubey P. (2023). Banana Peels as Bioactive Ingredient Systematic Review of Nutritional and Pharmacological Attributes. JFCN.

[63] Parisi C., Guerriero G. (2019). Antioxidative Defense and Fertility Ratein the Assessment of Reprotoxcity Risk Posed by Global Warming. Antioxid.

[64] Ashoka G.B., Manchanahally B.S. (2023). Antibacterial, antioxidant, and anticancer activities of *Penicillium citrinum* Thom. endophytic in *Jatropha heynei*. J. Appl. Pharm. Sci..

[65] Khan A., Chen H., Wan X., Tania M., Xu A., Chen F., Zhang D. (2013). Regulatory effects of resveratrol on antioxidant enzymes: A mechanism of growth inhibition and apoptosis induction in cancer cells. Mol. Cells.

[66] Sedlak L., Wojnar W., Zych M., Wyględowska-Promieńska D., Mrukwa-Kominek E., Kaczmarczyk-Sedlak I. (2018). Effect of Resveratrol, a Dietary-Derived Polyphenol, on the Oxidative Stress and Polyol Pathway in the Lens of Rats with Streptozotocin-Induced Diabetes. Nutrients.

[67] Banu S.K., Stanley J.A., Sivakumar K.K., Arosh J.A., Burghardt R.C. (2016). Resveratrol protects the ovary against chromium-toxicity by enhancing endogenous antioxidant enzymes and inhibiting metabolic clearance of estradiol. Toxicol. Appl. Pharmacol..

[68] Maleki M.H., Omidi F., Javanshir Z., Bagheri M., Tanhadoroodzani Z., Dastghaib S., Shams M., Akbari M., Dastghaib S. (2024). β-Hydroxybutyrate and melatonin suppress maladaptive UPR, excessive autophagy and pyroptosis in Aβ 1–42 and LPS-Induced SH-SY5Y cells. Mol. Biol. Rep..

[69] Hu X., Ma W., Zhang D., Tian Z., Yang Y., Huang Y., Hong Y. (2025). Application of Natural Antioxidants as Feed Additives in Aquaculture: A Review. Biology.

[70] Park J., Kil Y.S., Ryoo G.H., Jin C.H., Hong M.J., Kim J.B., Jung C.H., Nam J.W., Han A.R. (2023). Phytochemical profile and anti-inflammatory activity of the hull of γ-irradiated wheat mutant lines (*Triticum aestivum* L.). Front. Nutr..

[71] Lu Y., Wang K., Hu L. (2025). Advancements in delivery systems for dietary polyphenols in enhancing radioprotection effects: Challenges and opportunities. npj Sci. Food.

[72] Khan H., Ullah H., Castilho P.C.M.F., Gomila A.S., D’Onofrio G., Filosa R., Wang F., Nabavi S.M., Daglia M., Sanchez Silva A. (2020). Targeting NF-κB signaling pathway in cancer by dietary polyphenols. Crit. Rev. Food Sci. Nutr..

[73] Aqil F., Munagala R., Agrawal A.K., Jeyabalan J., Tyagi N., Rai S.N., Gupta R.C. (2021). Anthocyanidins inhibit growth and chemosensitize triple-negative breast cancer via the NF-κB signaling pathway. Cancers.

[74] Umahi-Ottah G., Obafemi F.A. (2023). A review of the effect of fertility agents (Herbs and drugs) on the hypothalamus. WJARR.

[75] Cote B., Elbarbry F., Bui F., Su J.W., Seo K., Nguyen A., Lee M., Rao D.A. (2022). Mechanistic Basis for the Role of Phytochemicals in Inflammation-Associated Chronic Diseases. Molecules.

[76] Akanda M.d.R., Kim M.J., Kim I.S., Ahn D., Tae H.J., Rahman M.M., Park Y.G., Seol J.W., Nam h.h., Choo B.K. (2018). Neuroprotective effects of *Sigesbeckia pubescens* extract on glutamate-induced oxidative stress in HT22 cells via downregulation of MAPK/caspase-3 pathways. Cell. Mol. Neurobiol..

[77] Alzokaky A.A., Al-Karmalawy A.A., Saleh M.A., Abdo W., Farage A.E., Belal A., Abourehab M.A., Antar S.A. (2023). Metformin ameliorates doxorubicin-induced cardiotoxicity targeting HMGB1/TLR4/NLRP3 signaling pathway in mice. Life Sci..

[78] Sadowska-Bartosz I., Bartosz G. (2015). Prevention of protein glycation by natural compounds. Molecules.

[79] Yazdani J., Dehzad M.J., Ommati M.M., Heidari R., Arabnezhad M.R., Hejazi N., Ahmadi A. (2025). The Effect of Banana (Musa nana Lour.) Peel Extract and Omega-3 on Biochemical and Histopathological Characteritics in Rat Model of Polycystic Ovary Syndrome. Int. J. Nutr. Sci..

[80] Taiwo A.M., Ige S.O., Babalola O.O. (2010). Assessments of possible gonadotoxic effect of lead on experimental male rabbits. Glob. Vet..

[81] Biswas N.M., Ghosh P.K. (2006). Protection of adrenal and male gonadal functions by androgen in lead-treated rats. Kathmandu Univ. Med. J..

[82] Daku A.B., Salisu A.I. (2016). Age-related effects of lead poisoning on sex hormones in adult male Wistar rats. J. Physiol. Pathophysiol..

[83] Riaz F., Khan U.A., Ayub M., Shaukat S. (2011). Protective role of ginger on lead induced derangement in plasma testosterone and LH levels of male sprague dawley rats. JAMC.

[84] El-Sayed Y.S., El-Neweshy M.S. (2010). Impact of lead toxicity on male rat reproduction at “hormonal and histopathological levels”. Toxicol. Environ. Chem..

[85] Gakunga N.J., Mugisha K., Owiny D., Waako P. (2014). Effects of Crude Aqueous Leaf Extracts of *Citropsis Articulata* and *Mystroxylon Aethiopicum* on Sex Hormone Levels In Male Albino Rats. IJPS.

[86] Gerriero G., Ferro R., Ciarcia G. (2005). Correlations between plasma levels of sex steroids and spermatogenesis during the sexual cycle of the chub, *Leuciscus cephalus* L. (Pisces: Cyprinidae). Zool. Stud..

[87] Guerriero G. (2007). Seasonal steroids variations and maturity stages in the female chub, *Leuciscus cephalus* L.(Pisces, Cyprinidae). Ital. J. Zool..

[88] Guerriero G., Prins G.S., Birch L., Ciarcia G. (2005). Neurodistribution of androgen receptor immunoreactivity in the male frog, *Rana esculenta*. Ann. N. Y. Acad. Sci..

